# Supplementary material for: PRAME Is a Novel Target of Tumor-Intrinsic Gas6/Axl Activation and Promotes Cancer Cell Invasion in Hepatocellular Carcinoma
Source: Cancers (Basel). 2023 Apr 22;15(9):2415. doi: 10.3390/cancers15092415 (PMC10177160; doi:10.3390/cancers15092415)
Supplement: Supplementary file 1 [file cancers-15-02415-s001.zip › cancers-2333836-supplementary.pptx]

## Slide 1
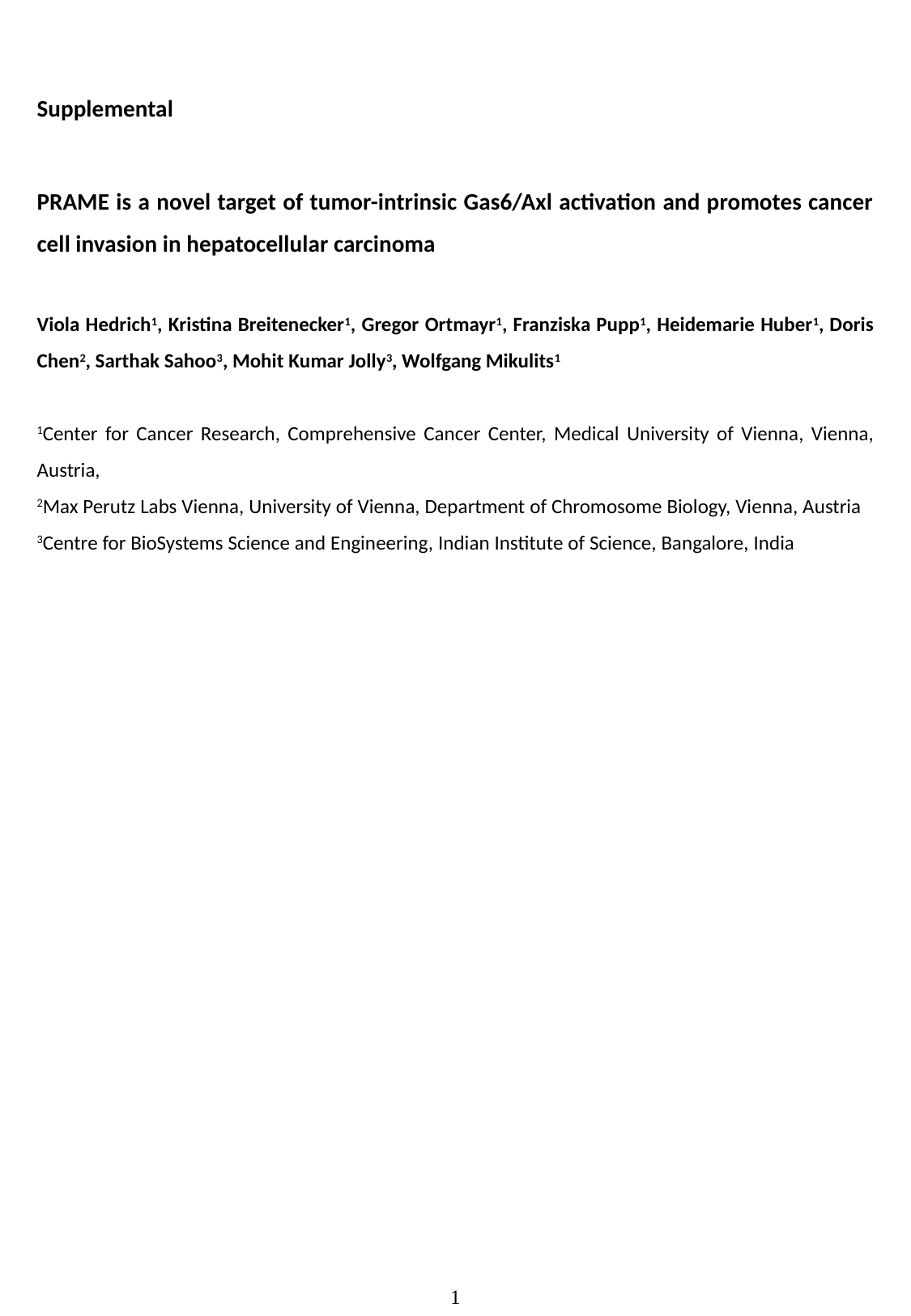

Supplemental
PRAME is a novel target of tumor-intrinsic Gas6/Axl activation and promotes cancer cell invasion in hepatocellular carcinoma
Viola Hedrich1, Kristina Breitenecker1, Gregor Ortmayr1, Franziska Pupp1, Heidemarie Huber1, Doris Chen2, Sarthak Sahoo3, Mohit Kumar Jolly3, Wolfgang Mikulits1
1Center for Cancer Research, Comprehensive Cancer Center, Medical University of Vienna, Vienna, Austria,
2Max Perutz Labs Vienna, University of Vienna, Department of Chromosome Biology, Vienna, Austria
3Centre for BioSystems Science and Engineering, Indian Institute of Science, Bangalore, India
1

## Slide 2
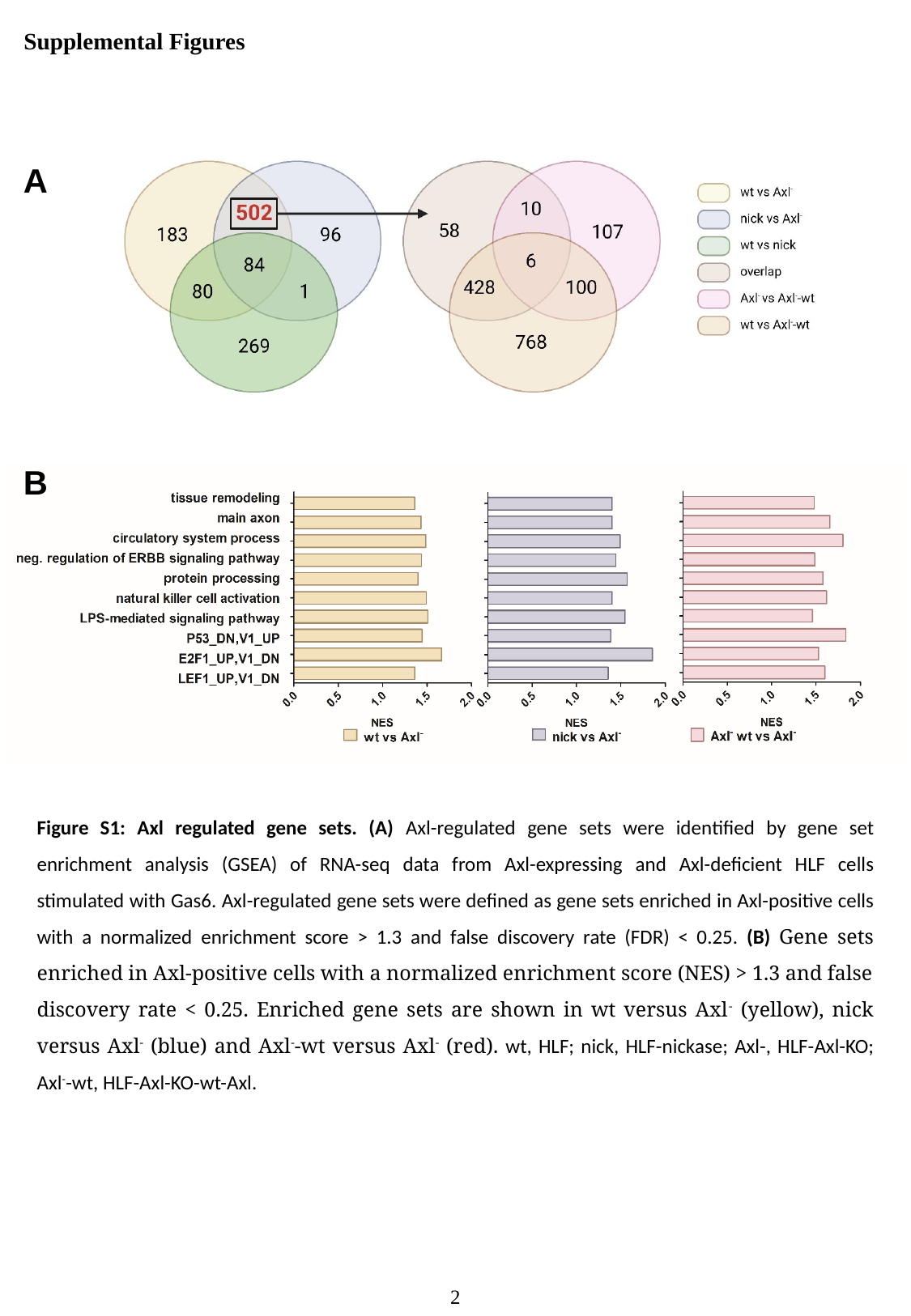

Supplemental Figures
A
B
Figure S1: Axl regulated gene sets. (A) Axl-regulated gene sets were identified by gene set enrichment analysis (GSEA) of RNA-seq data from Axl-expressing and Axl-deficient HLF cells stimulated with Gas6. Axl-regulated gene sets were defined as gene sets enriched in Axl-positive cells with a normalized enrichment score > 1.3 and false discovery rate (FDR) < 0.25. (B) Gene sets enriched in Axl-positive cells with a normalized enrichment score (NES) > 1.3 and false discovery rate < 0.25. Enriched gene sets are shown in wt versus Axl- (yellow), nick versus Axl- (blue) and Axl--wt versus Axl- (red). wt, HLF; nick, HLF-nickase; Axl-, HLF-Axl-KO; Axl--wt, HLF-Axl-KO-wt-Axl.
2

## Slide 3
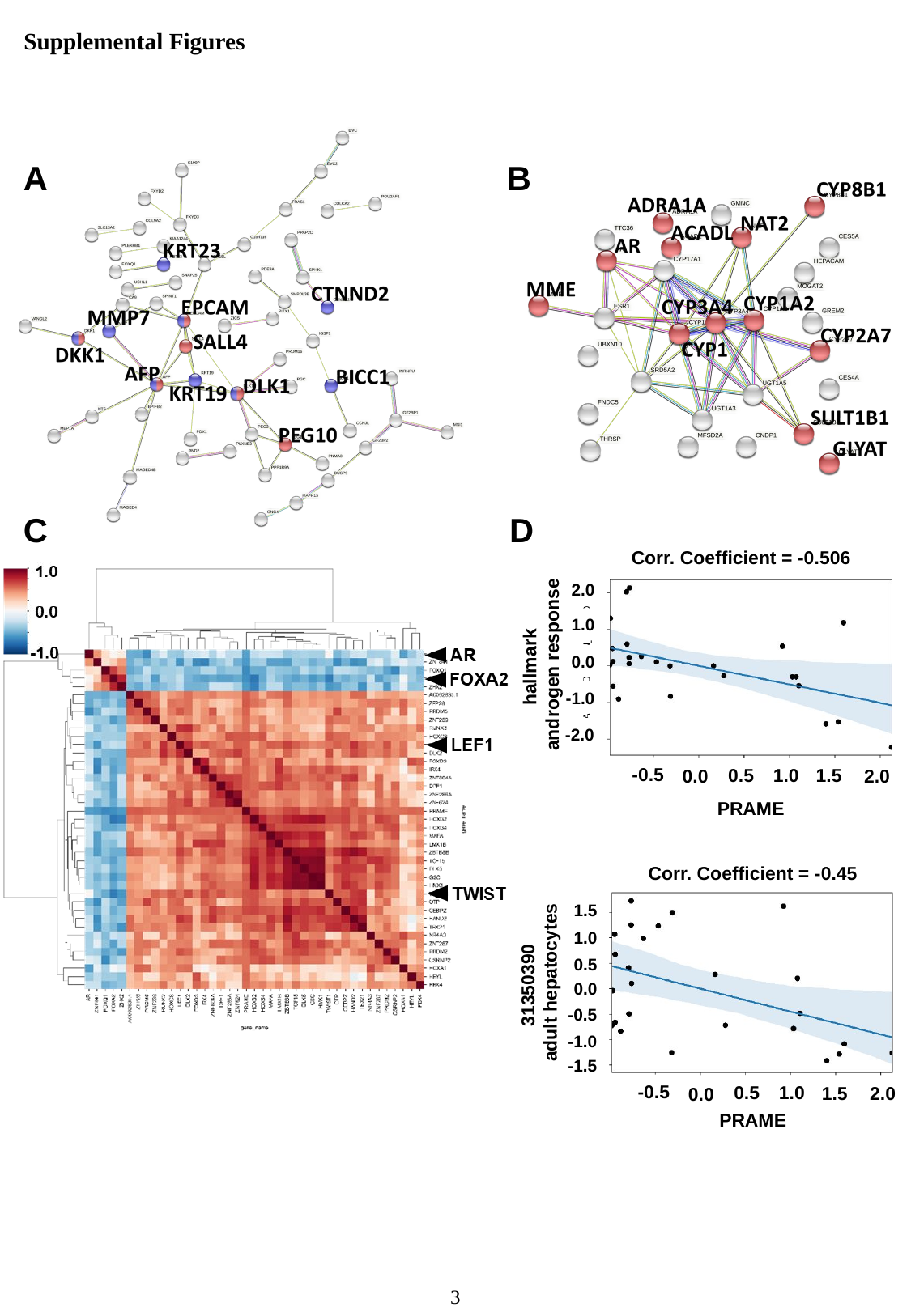

Supplemental Figures
A
B
C
D
Corr. Coefficient = -0.506
2.0
1.0
0.0
-1.0
-2.0
hallmark
androgen response
-0.5
1.0
0.5
1.5
2.0
0.0
PRAME
Corr. Coefficient = -0.45
1.5
1.0
0.5
0.0
-0.5
-1.0
-1.5
31350390
adult hepatocytes
-0.5
1.0
0.5
1.5
2.0
0.0
PRAME
3

## Slide 4
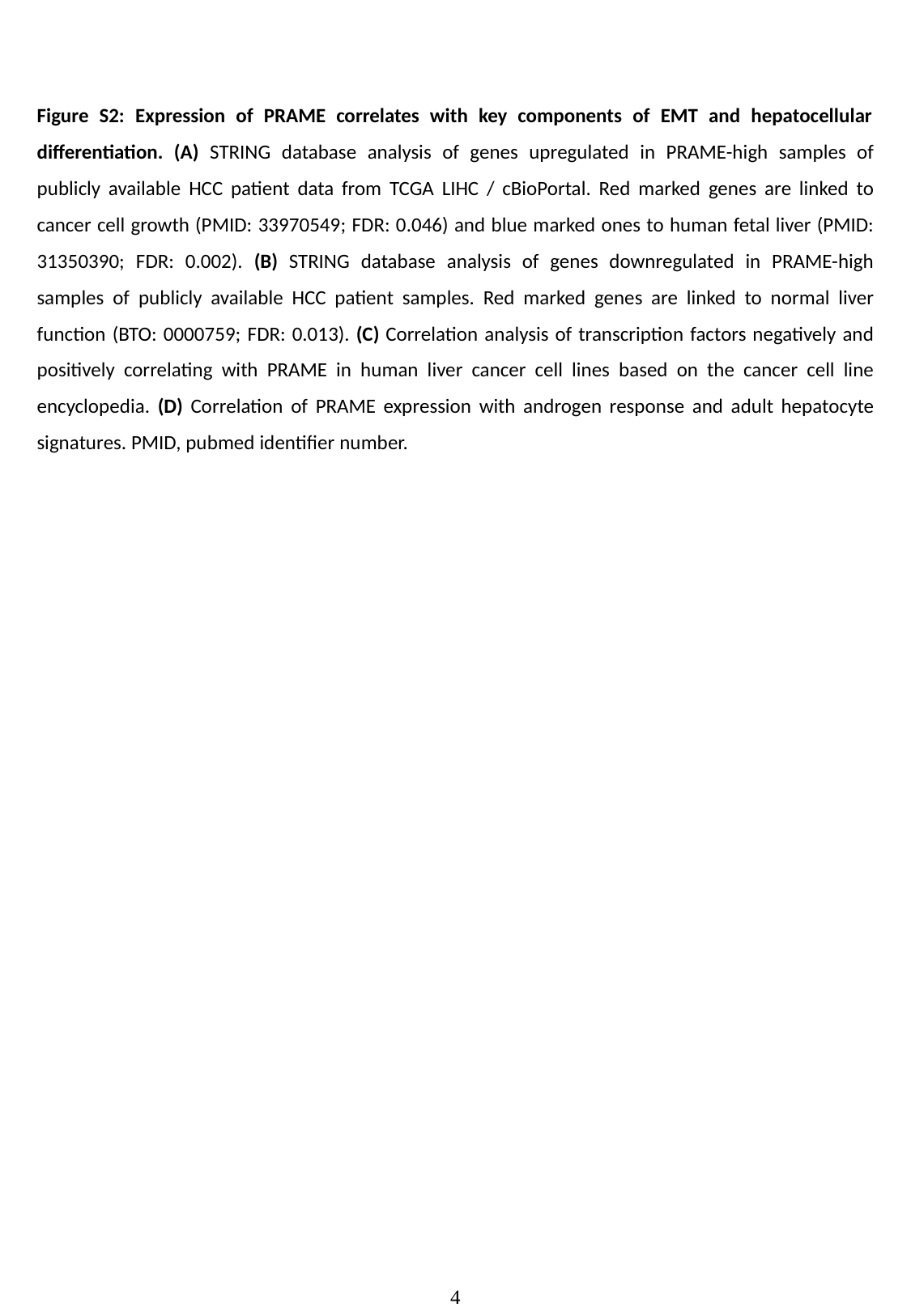

Figure S2: Expression of PRAME correlates with key components of EMT and hepatocellular differentiation. (A) STRING database analysis of genes upregulated in PRAME-high samples of publicly available HCC patient data from TCGA LIHC / cBioPortal. Red marked genes are linked to cancer cell growth (PMID: 33970549; FDR: 0.046) and blue marked ones to human fetal liver (PMID: 31350390; FDR: 0.002). (B) STRING database analysis of genes downregulated in PRAME-high samples of publicly available HCC patient samples. Red marked genes are linked to normal liver function (BTO: 0000759; FDR: 0.013). (C) Correlation analysis of transcription factors negatively and positively correlating with PRAME in human liver cancer cell lines based on the cancer cell line encyclopedia. (D) Correlation of PRAME expression with androgen response and adult hepatocyte signatures. PMID, pubmed identifier number.
4

## Slide 5
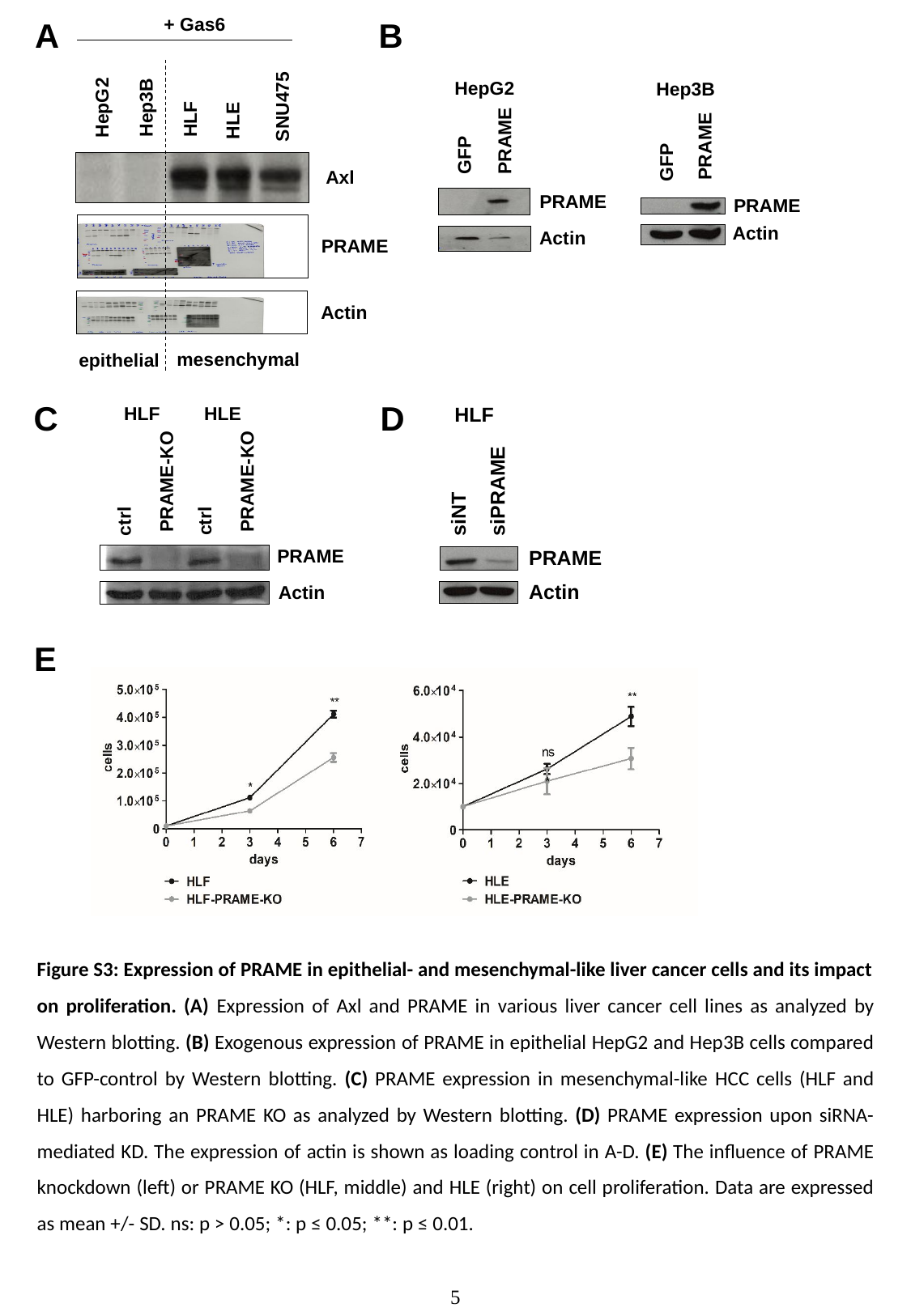

+ Gas6
SNU475
Hep3B
HepG2
HLF
HLE
mesenchymal
epithelial
Axl
PRAME
Actin
A
B
HepG2
PRAME
GFP
PRAME
Actin
Hep3B
PRAME
GFP
PRAME
Actin
C
D
HLF
siPRAME
siNT
PRAME
Actin
HLE
HLF
PRAME-KO
PRAME-KO
ctrl
ctrl
PRAME
Actin
E
Figure S3: Expression of PRAME in epithelial- and mesenchymal-like liver cancer cells and its impact on proliferation. (A) Expression of Axl and PRAME in various liver cancer cell lines as analyzed by Western blotting. (B) Exogenous expression of PRAME in epithelial HepG2 and Hep3B cells compared to GFP-control by Western blotting. (C) PRAME expression in mesenchymal-like HCC cells (HLF and HLE) harboring an PRAME KO as analyzed by Western blotting. (D) PRAME expression upon siRNA-mediated KD. The expression of actin is shown as loading control in A-D. (E) The influence of PRAME knockdown (left) or PRAME KO (HLF, middle) and HLE (right) on cell proliferation. Data are expressed as mean +/- SD. ns: p > 0.05; *: p ≤ 0.05; **: p ≤ 0.01.
5

## Slide 6
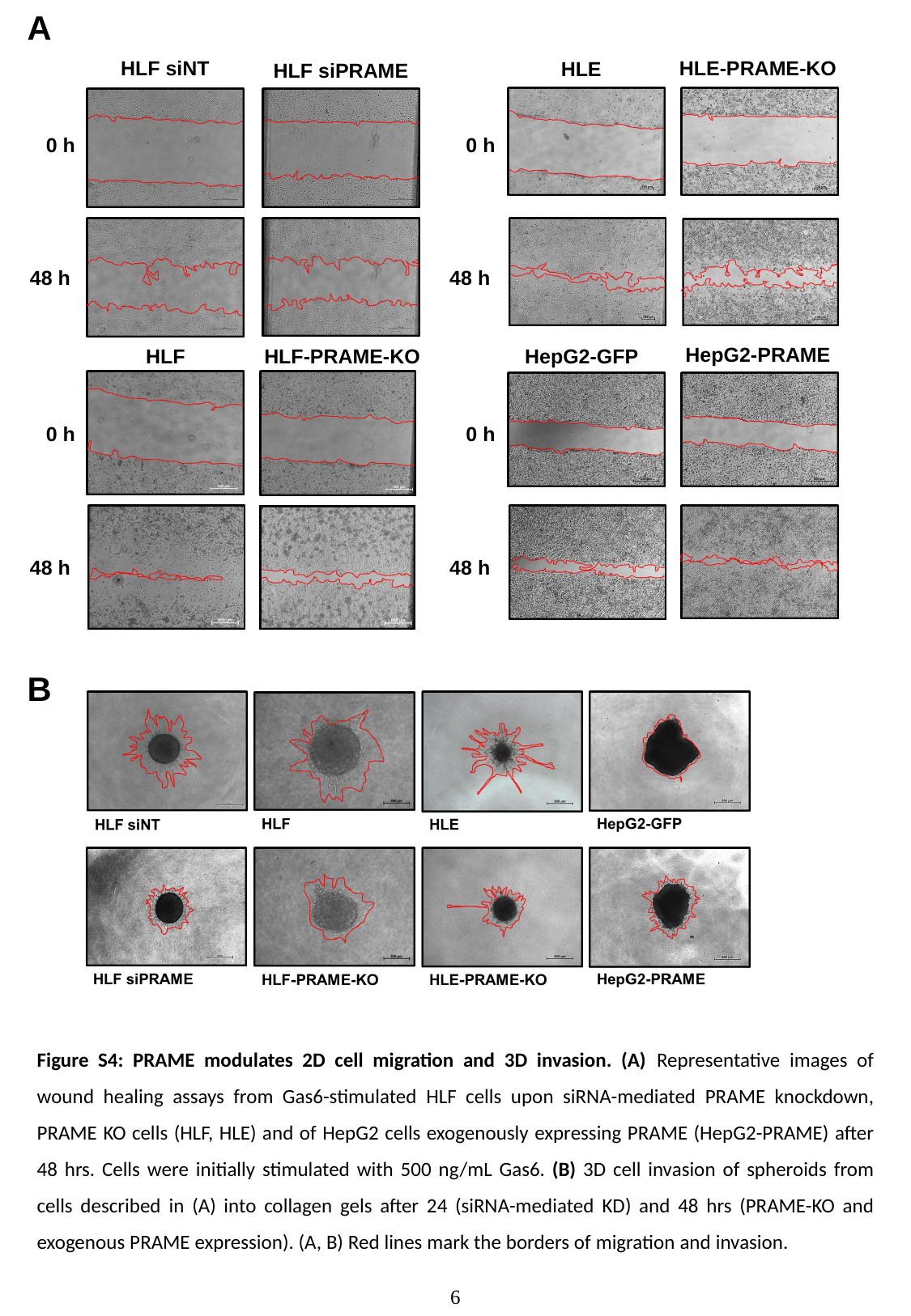

A
HLF siNT
HLF siPRAME
0 h
48 h
HLE-PRAME-KO
HLE
0 h
48 h
HepG2-PRAME
HepG2-GFP
0 h
48 h
HLF
HLF-PRAME-KO
0 h
48 h
B
Figure S4: PRAME modulates 2D cell migration and 3D invasion. (A) Representative images of wound healing assays from Gas6-stimulated HLF cells upon siRNA-mediated PRAME knockdown, PRAME KO cells (HLF, HLE) and of HepG2 cells exogenously expressing PRAME (HepG2-PRAME) after 48 hrs. Cells were initially stimulated with 500 ng/mL Gas6. (B) 3D cell invasion of spheroids from cells described in (A) into collagen gels after 24 (siRNA-mediated KD) and 48 hrs (PRAME-KO and exogenous PRAME expression). (A, B) Red lines mark the borders of migration and invasion.
6

## Slide 7
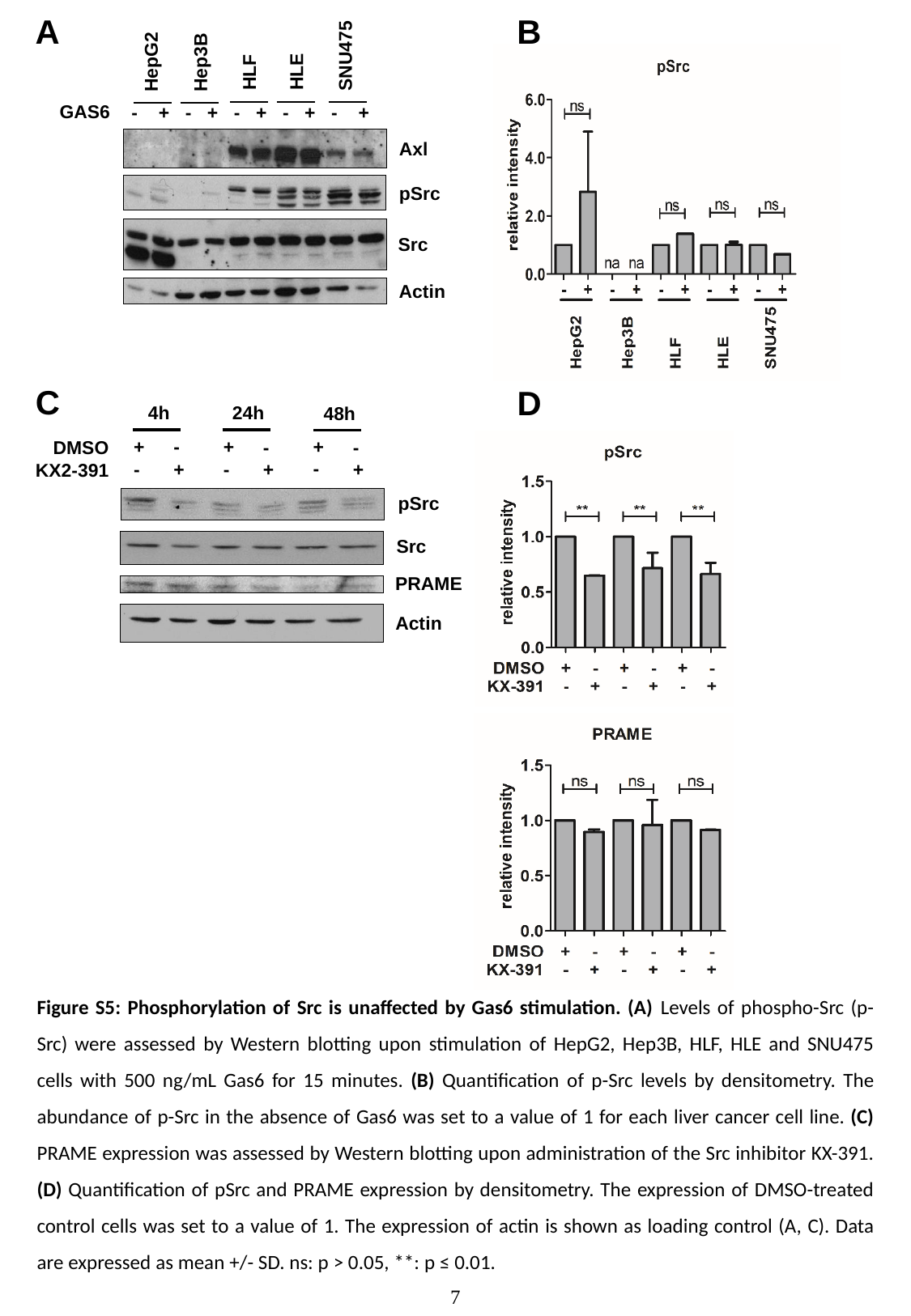

A
SNU475
HLF
HLE
Hep3B
HepG2
GAS6
- + - + - + - + - +
Axl
pSrc
Src
Actin
B
C
D
4h
+
-
-
+
DMSO
KX2-391
pSrc
Src
PRAME
Actin
24h
48h
+
-
+
-
-
+
-
+
Figure S5: Phosphorylation of Src is unaffected by Gas6 stimulation. (A) Levels of phospho-Src (p-Src) were assessed by Western blotting upon stimulation of HepG2, Hep3B, HLF, HLE and SNU475 cells with 500 ng/mL Gas6 for 15 minutes. (B) Quantification of p-Src levels by densitometry. The abundance of p-Src in the absence of Gas6 was set to a value of 1 for each liver cancer cell line. (C) PRAME expression was assessed by Western blotting upon administration of the Src inhibitor KX-391. (D) Quantification of pSrc and PRAME expression by densitometry. The expression of DMSO-treated control cells was set to a value of 1. The expression of actin is shown as loading control (A, C). Data are expressed as mean +/- SD. ns: p > 0.05, **: p ≤ 0.01.
7

## Slide 8
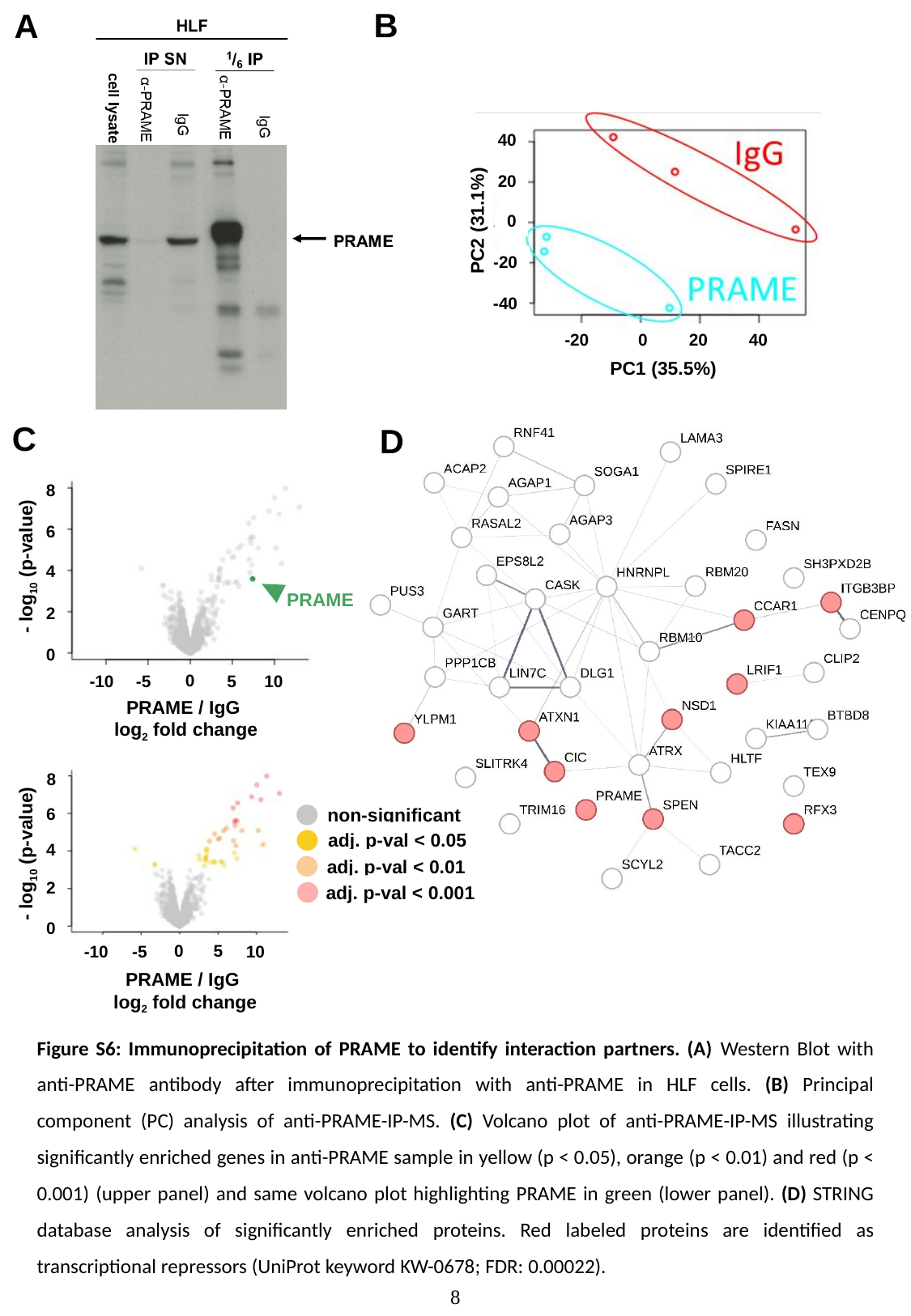

B
A
40
20
0
-20
-40
PC2 (31.1%)
-20
0
40
20
PC1 (35.5%)
C
D
8
6
4
2
0
- log10 (p-value)
PRAME
0
5
-10
10
-5
PRAME / IgG
log2 fold change
8
6
4
2
0
non-significant
adj. p-val < 0.05
adj. p-val < 0.01
adj. p-val < 0.001
- log10 (p-value)
0
5
-10
10
-5
PRAME / IgG
log2 fold change
Figure S6: Immunoprecipitation of PRAME to identify interaction partners. (A) Western Blot with anti-PRAME antibody after immunoprecipitation with anti-PRAME in HLF cells. (B) Principal component (PC) analysis of anti-PRAME-IP-MS. (C) Volcano plot of anti-PRAME-IP-MS illustrating significantly enriched genes in anti-PRAME sample in yellow (p < 0.05), orange (p < 0.01) and red (p < 0.001) (upper panel) and same volcano plot highlighting PRAME in green (lower panel). (D) STRING database analysis of significantly enriched proteins. Red labeled proteins are identified as transcriptional repressors (UniProt keyword KW-0678; FDR: 0.00022).
8

## Slide 9
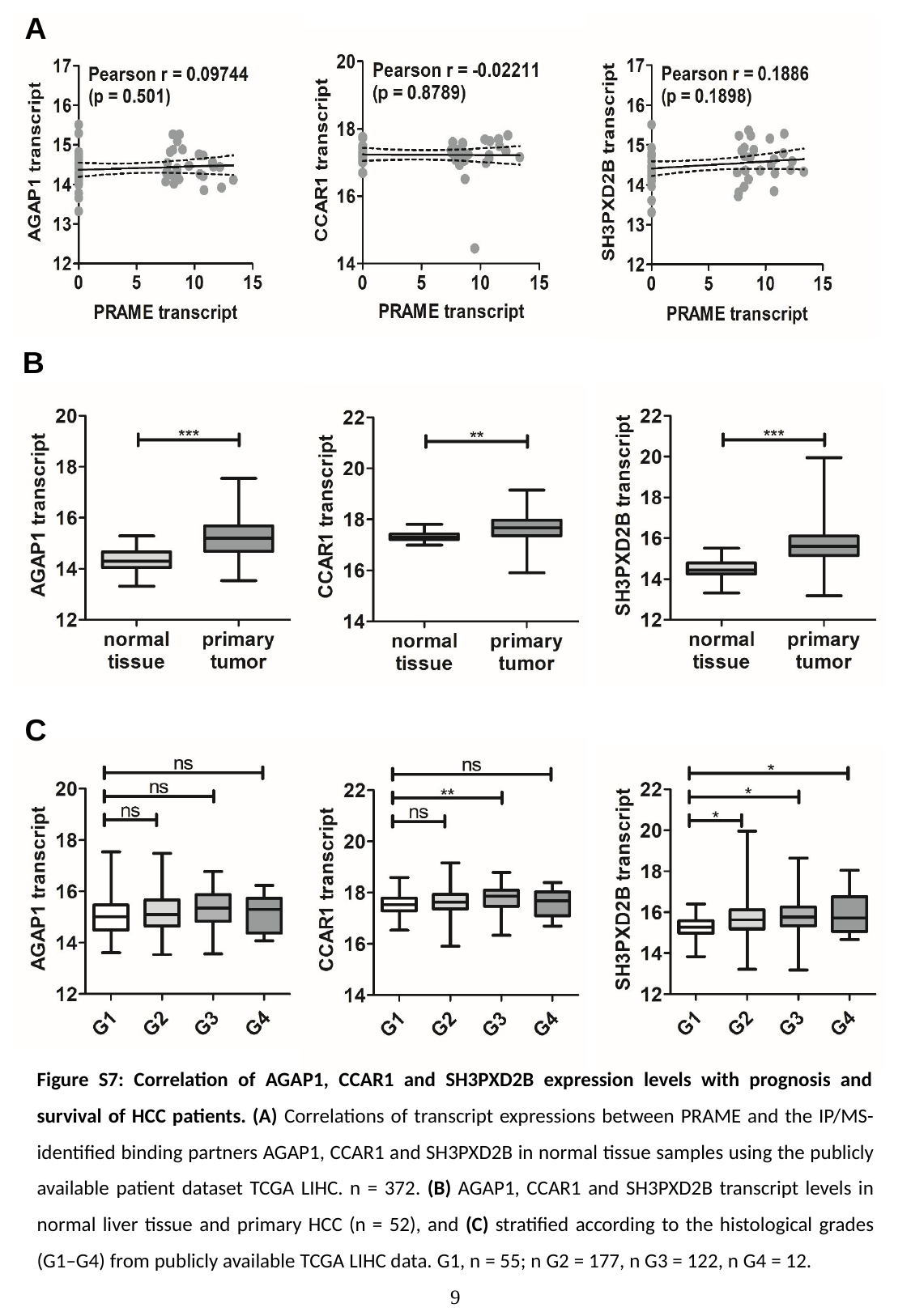

A
B
C
Figure S7: Correlation of AGAP1, CCAR1 and SH3PXD2B expression levels with prognosis and survival of HCC patients. (A) Correlations of transcript expressions between PRAME and the IP/MS-identified binding partners AGAP1, CCAR1 and SH3PXD2B in normal tissue samples using the publicly available patient dataset TCGA LIHC. n = 372. (B) AGAP1, CCAR1 and SH3PXD2B transcript levels in normal liver tissue and primary HCC (n = 52), and (C) stratified according to the histological grades (G1–G4) from publicly available TCGA LIHC data. G1, n = 55; n G2 = 177, n G3 = 122, n G4 = 12.
9

## Slide 10
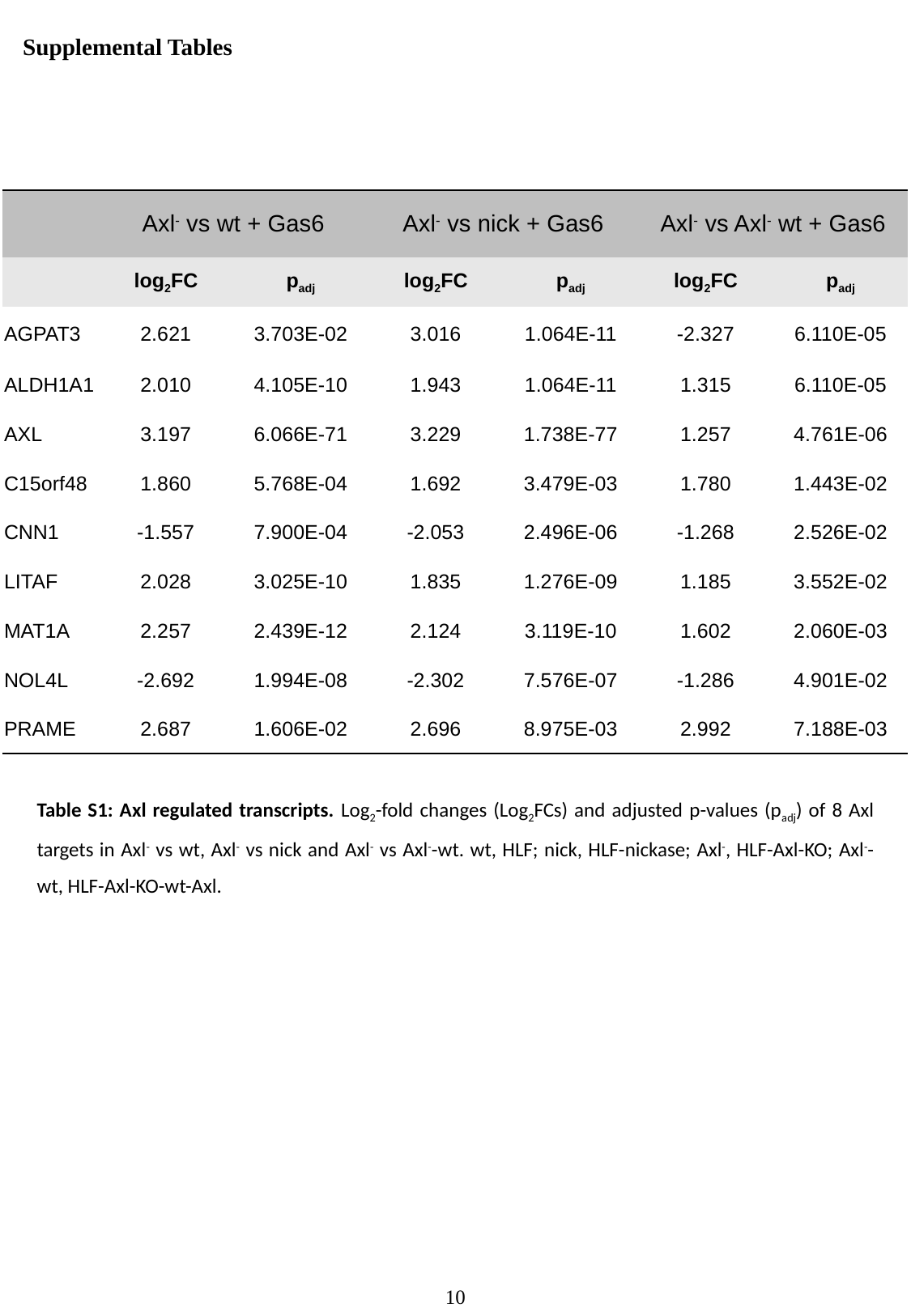

Supplemental Tables
| | Axl- vs wt + Gas6 | | Axl- vs nick + Gas6 | | Axl- vs Axl- wt + Gas6 | |
| --- | --- | --- | --- | --- | --- | --- |
| | log2FC | padj | log2FC | padj | log2FC | padj |
| AGPAT3 | 2.621 | 3.703E-02 | 3.016 | 1.064E-11 | -2.327 | 6.110E-05 |
| ALDH1A1 | 2.010 | 4.105E-10 | 1.943 | 1.064E-11 | 1.315 | 6.110E-05 |
| AXL | 3.197 | 6.066E-71 | 3.229 | 1.738E-77 | 1.257 | 4.761E-06 |
| C15orf48 | 1.860 | 5.768E-04 | 1.692 | 3.479E-03 | 1.780 | 1.443E-02 |
| CNN1 | -1.557 | 7.900E-04 | -2.053 | 2.496E-06 | -1.268 | 2.526E-02 |
| LITAF | 2.028 | 3.025E-10 | 1.835 | 1.276E-09 | 1.185 | 3.552E-02 |
| MAT1A | 2.257 | 2.439E-12 | 2.124 | 3.119E-10 | 1.602 | 2.060E-03 |
| NOL4L | -2.692 | 1.994E-08 | -2.302 | 7.576E-07 | -1.286 | 4.901E-02 |
| PRAME | 2.687 | 1.606E-02 | 2.696 | 8.975E-03 | 2.992 | 7.188E-03 |
Table S1: Axl regulated transcripts. Log2-fold changes (Log2FCs) and adjusted p-values (padj) of 8 Axl targets in Axl- vs wt, Axl- vs nick and Axl- vs Axl--wt. wt, HLF; nick, HLF-nickase; Axl-, HLF-Axl-KO; Axl--wt, HLF-Axl-KO-wt-Axl.
10

## Slide 11
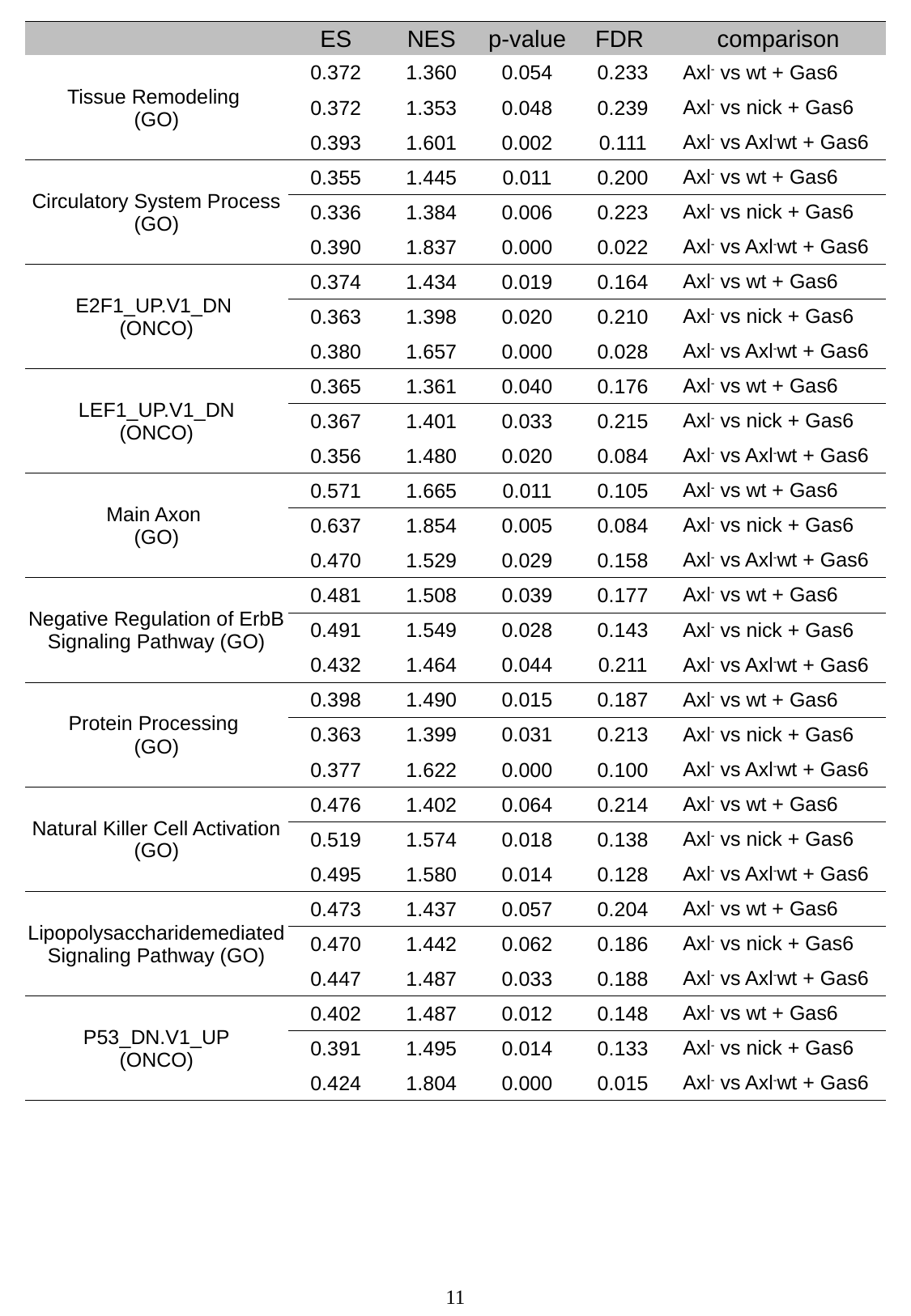

| | ES | NES | p-value | FDR | comparison |
| --- | --- | --- | --- | --- | --- |
| Tissue Remodeling (GO) | 0.372 | 1.360 | 0.054 | 0.233 | Axl- vs wt + Gas6 |
| | 0.372 | 1.353 | 0.048 | 0.239 | Axl- vs nick + Gas6 |
| | 0.393 | 1.601 | 0.002 | 0.111 | Axl- vs Axl-wt + Gas6 |
| Circulatory System Process (GO) | 0.355 | 1.445 | 0.011 | 0.200 | Axl- vs wt + Gas6 |
| | 0.336 | 1.384 | 0.006 | 0.223 | Axl- vs nick + Gas6 |
| | 0.390 | 1.837 | 0.000 | 0.022 | Axl- vs Axl-wt + Gas6 |
| E2F1\_UP.V1\_DN (ONCO) | 0.374 | 1.434 | 0.019 | 0.164 | Axl- vs wt + Gas6 |
| | 0.363 | 1.398 | 0.020 | 0.210 | Axl- vs nick + Gas6 |
| | 0.380 | 1.657 | 0.000 | 0.028 | Axl- vs Axl-wt + Gas6 |
| LEF1\_UP.V1\_DN (ONCO) | 0.365 | 1.361 | 0.040 | 0.176 | Axl- vs wt + Gas6 |
| | 0.367 | 1.401 | 0.033 | 0.215 | Axl- vs nick + Gas6 |
| | 0.356 | 1.480 | 0.020 | 0.084 | Axl- vs Axl-wt + Gas6 |
| Main Axon (GO) | 0.571 | 1.665 | 0.011 | 0.105 | Axl- vs wt + Gas6 |
| | 0.637 | 1.854 | 0.005 | 0.084 | Axl- vs nick + Gas6 |
| | 0.470 | 1.529 | 0.029 | 0.158 | Axl- vs Axl-wt + Gas6 |
| Negative Regulation of ErbB Signaling Pathway (GO) | 0.481 | 1.508 | 0.039 | 0.177 | Axl- vs wt + Gas6 |
| | 0.491 | 1.549 | 0.028 | 0.143 | Axl- vs nick + Gas6 |
| | 0.432 | 1.464 | 0.044 | 0.211 | Axl- vs Axl-wt + Gas6 |
| Protein Processing (GO) | 0.398 | 1.490 | 0.015 | 0.187 | Axl- vs wt + Gas6 |
| | 0.363 | 1.399 | 0.031 | 0.213 | Axl- vs nick + Gas6 |
| | 0.377 | 1.622 | 0.000 | 0.100 | Axl- vs Axl-wt + Gas6 |
| Natural Killer Cell Activation (GO) | 0.476 | 1.402 | 0.064 | 0.214 | Axl- vs wt + Gas6 |
| | 0.519 | 1.574 | 0.018 | 0.138 | Axl- vs nick + Gas6 |
| | 0.495 | 1.580 | 0.014 | 0.128 | Axl- vs Axl-wt + Gas6 |
| Lipopolysaccharidemediated Signaling Pathway (GO) | 0.473 | 1.437 | 0.057 | 0.204 | Axl- vs wt + Gas6 |
| | 0.470 | 1.442 | 0.062 | 0.186 | Axl- vs nick + Gas6 |
| | 0.447 | 1.487 | 0.033 | 0.188 | Axl- vs Axl-wt + Gas6 |
| P53\_DN.V1\_UP (ONCO) | 0.402 | 1.487 | 0.012 | 0.148 | Axl- vs wt + Gas6 |
| | 0.391 | 1.495 | 0.014 | 0.133 | Axl- vs nick + Gas6 |
| | 0.424 | 1.804 | 0.000 | 0.015 | Axl- vs Axl-wt + Gas6 |
11

## Slide 12
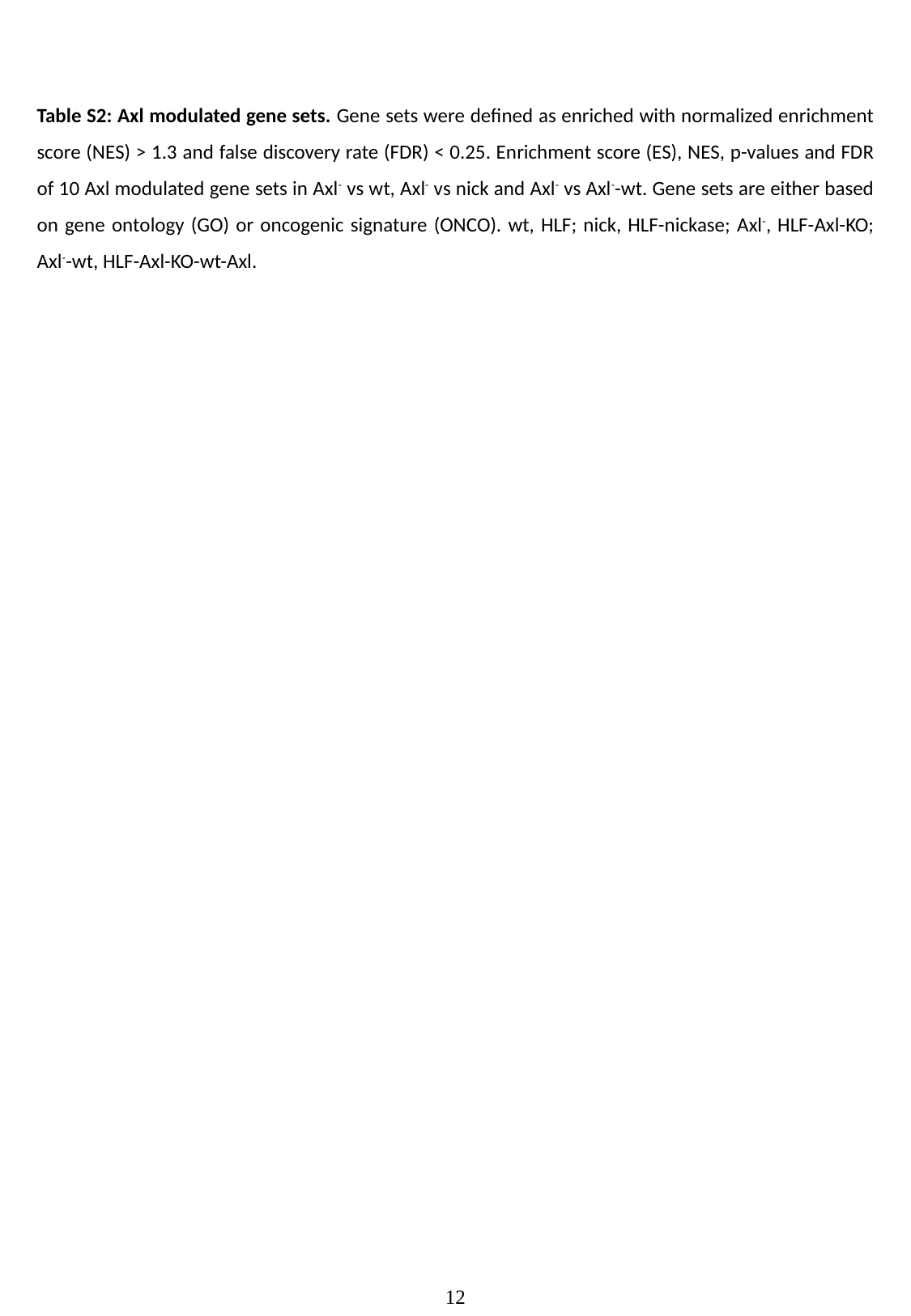

Table S2: Axl modulated gene sets. Gene sets were defined as enriched with normalized enrichment score (NES) > 1.3 and false discovery rate (FDR) < 0.25. Enrichment score (ES), NES, p-values and FDR of 10 Axl modulated gene sets in Axl- vs wt, Axl- vs nick and Axl- vs Axl--wt. Gene sets are either based on gene ontology (GO) or oncogenic signature (ONCO). wt, HLF; nick, HLF-nickase; Axl-, HLF-Axl-KO; Axl--wt, HLF-Axl-KO-wt-Axl.
12

## Slide 13
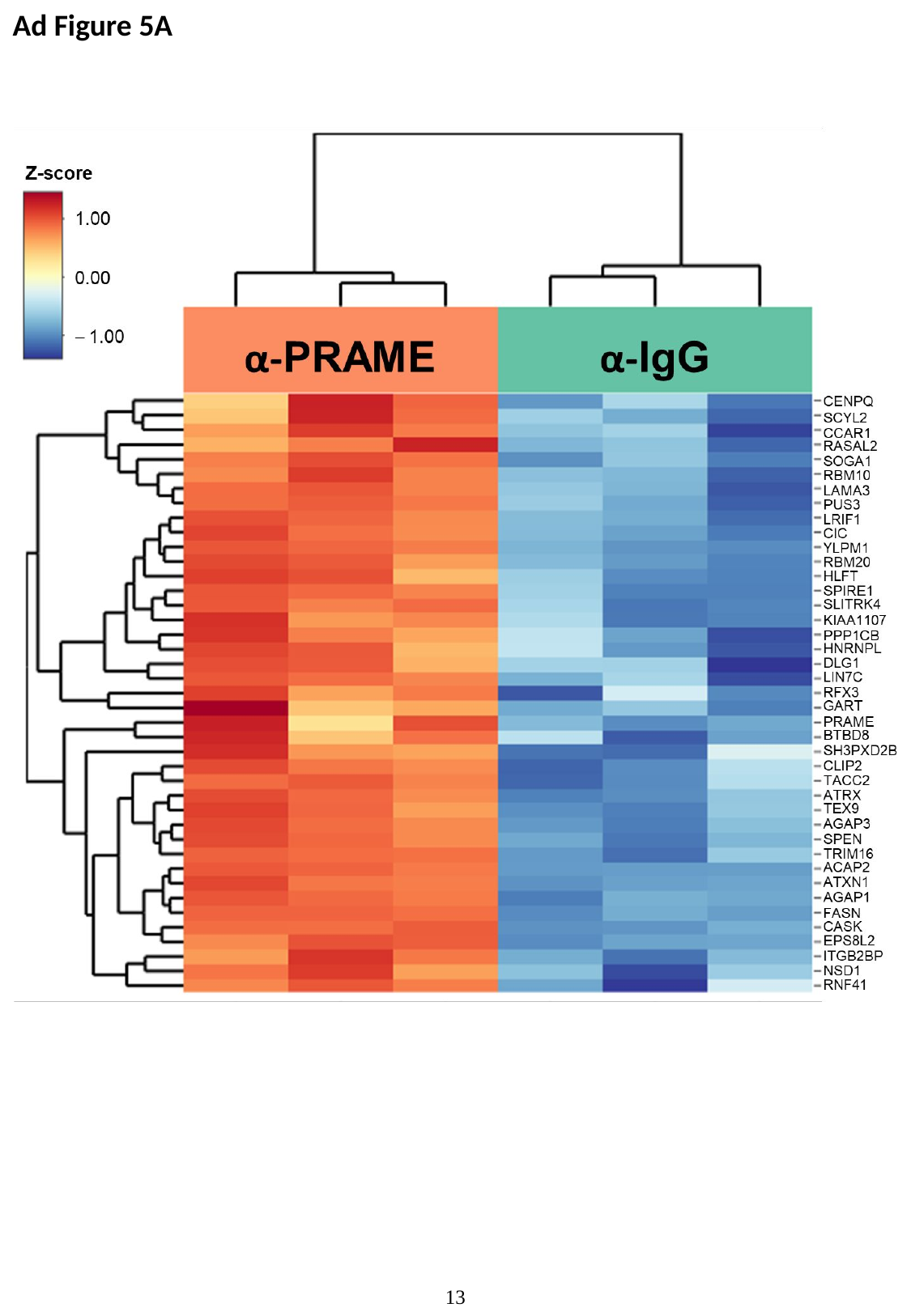

Ad Figure 5A
13

## Slide 14
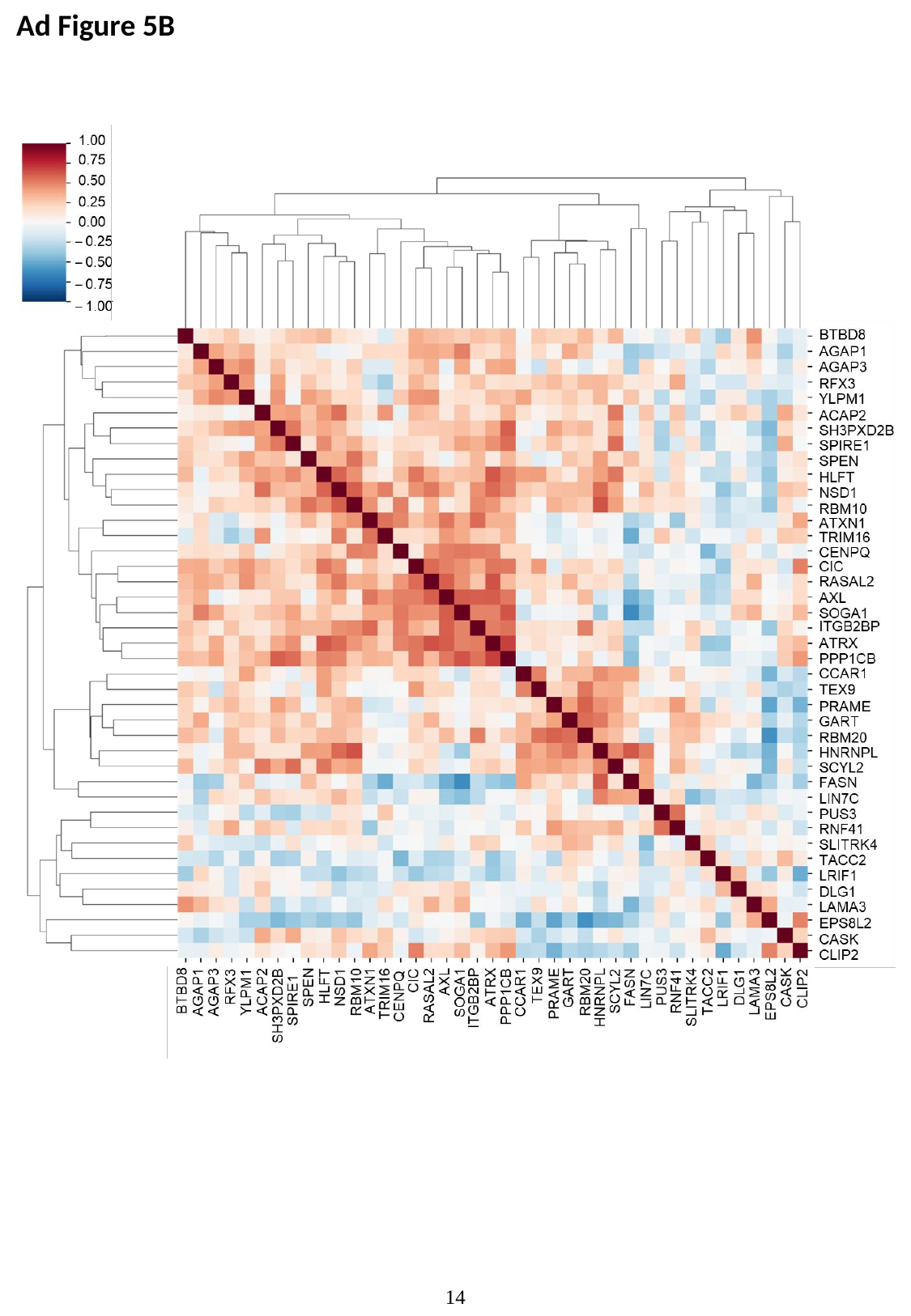

Ad Figure 5B
14

## Slide 15
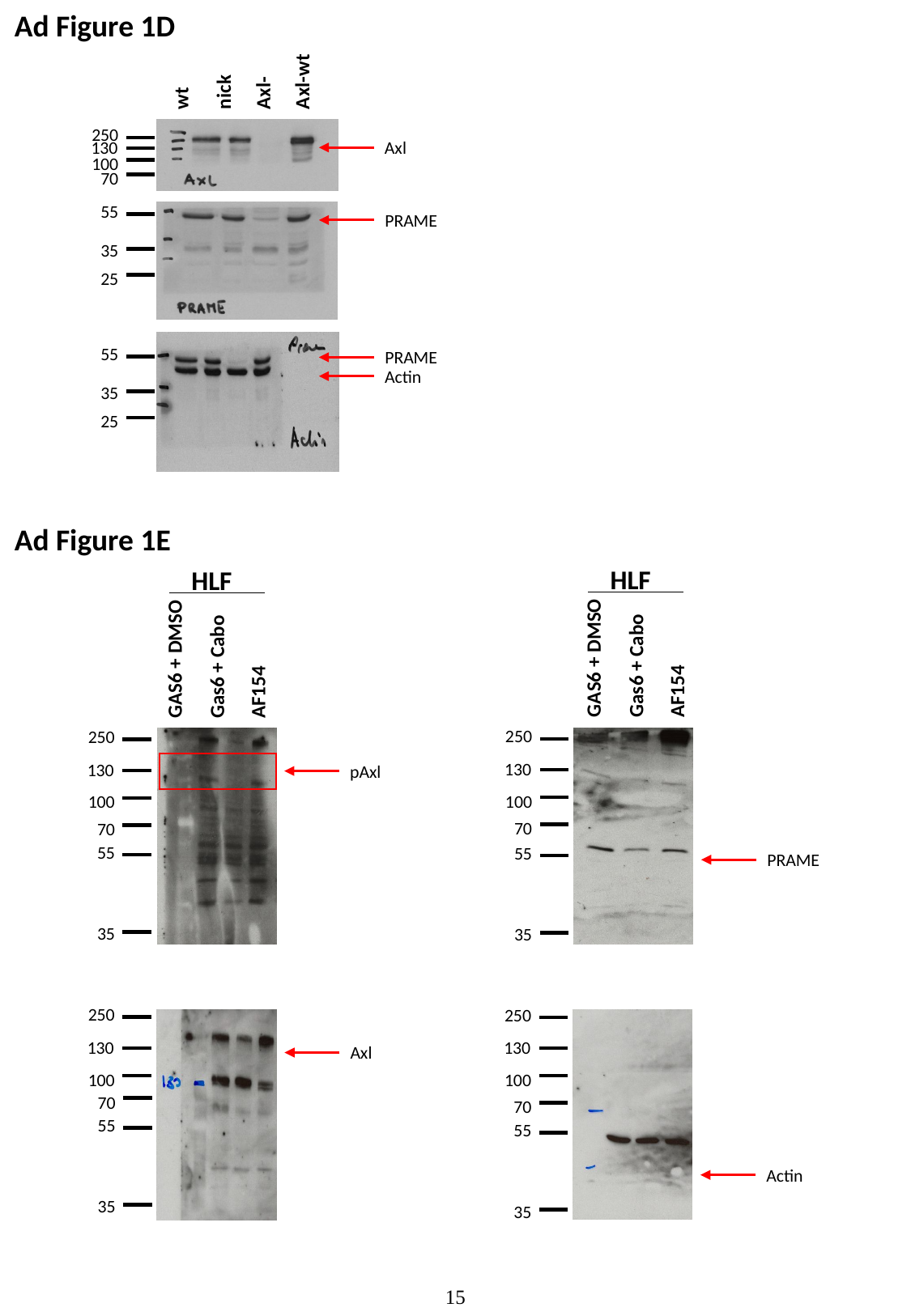

Ad Figure 1D
Axl-wt
nick
Axl-
wt
250
Axl
130
100
70
55
PRAME
35
25
55
PRAME
Actin
35
25
Ad Figure 1E
HLF
GAS6 + DMSO
Gas6 + Cabo
AF154
250
130
100
70
55
PRAME
35
HLF
GAS6 + DMSO
Gas6 + Cabo
AF154
250
130
pAxl
100
70
55
35
250
130
Axl
100
70
55
35
250
130
100
70
55
35
Actin
15

## Slide 16
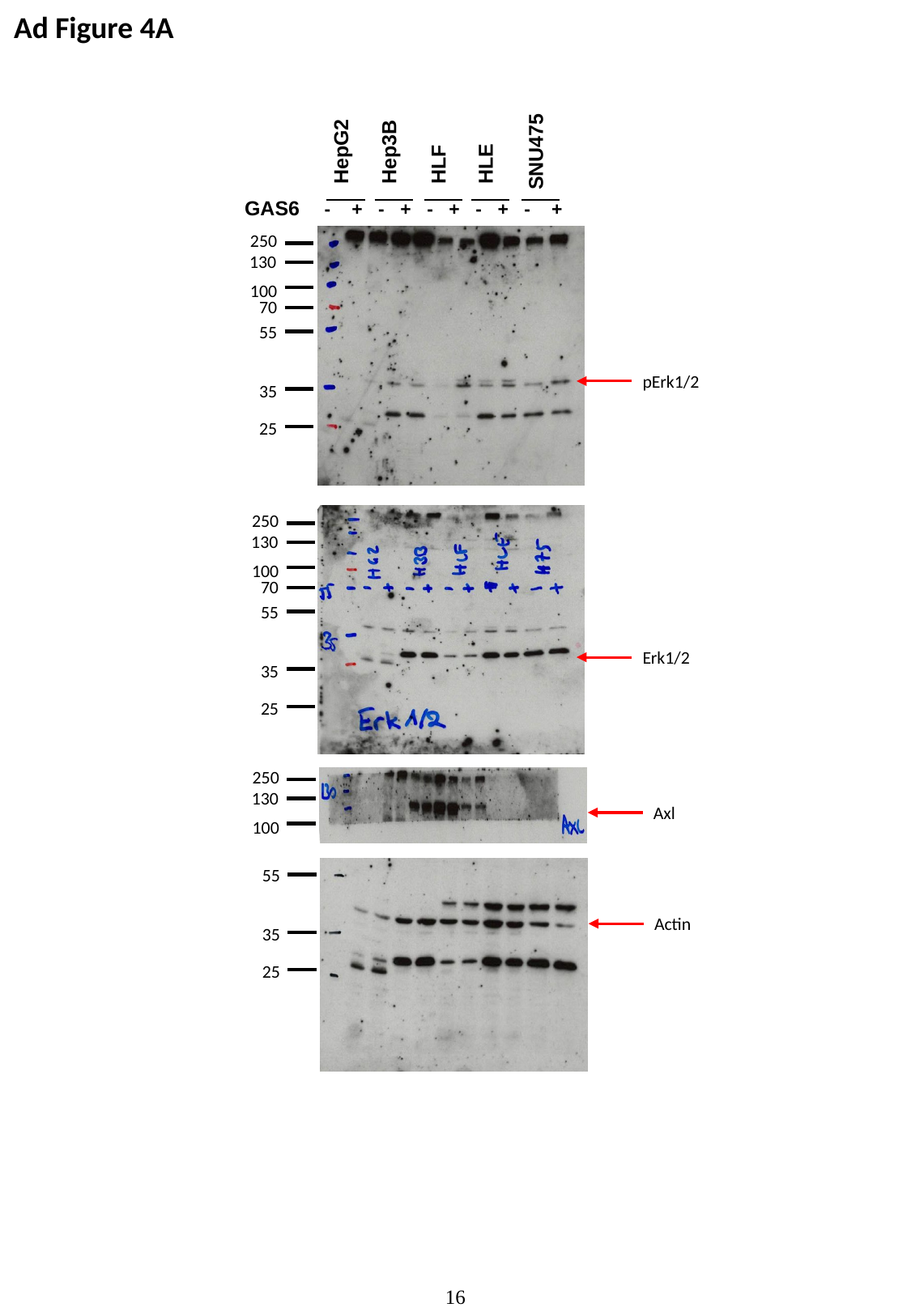

Ad Figure 4A
HepG2
Hep3B
HLF
HLE
GAS6
- + - + - + - + - +
SNU475
250
130
100
70
55
pErk1/2
35
25
250
130
100
70
55
Erk1/2
35
25
250
130
Axl
100
55
Actin
35
25
16

## Slide 17
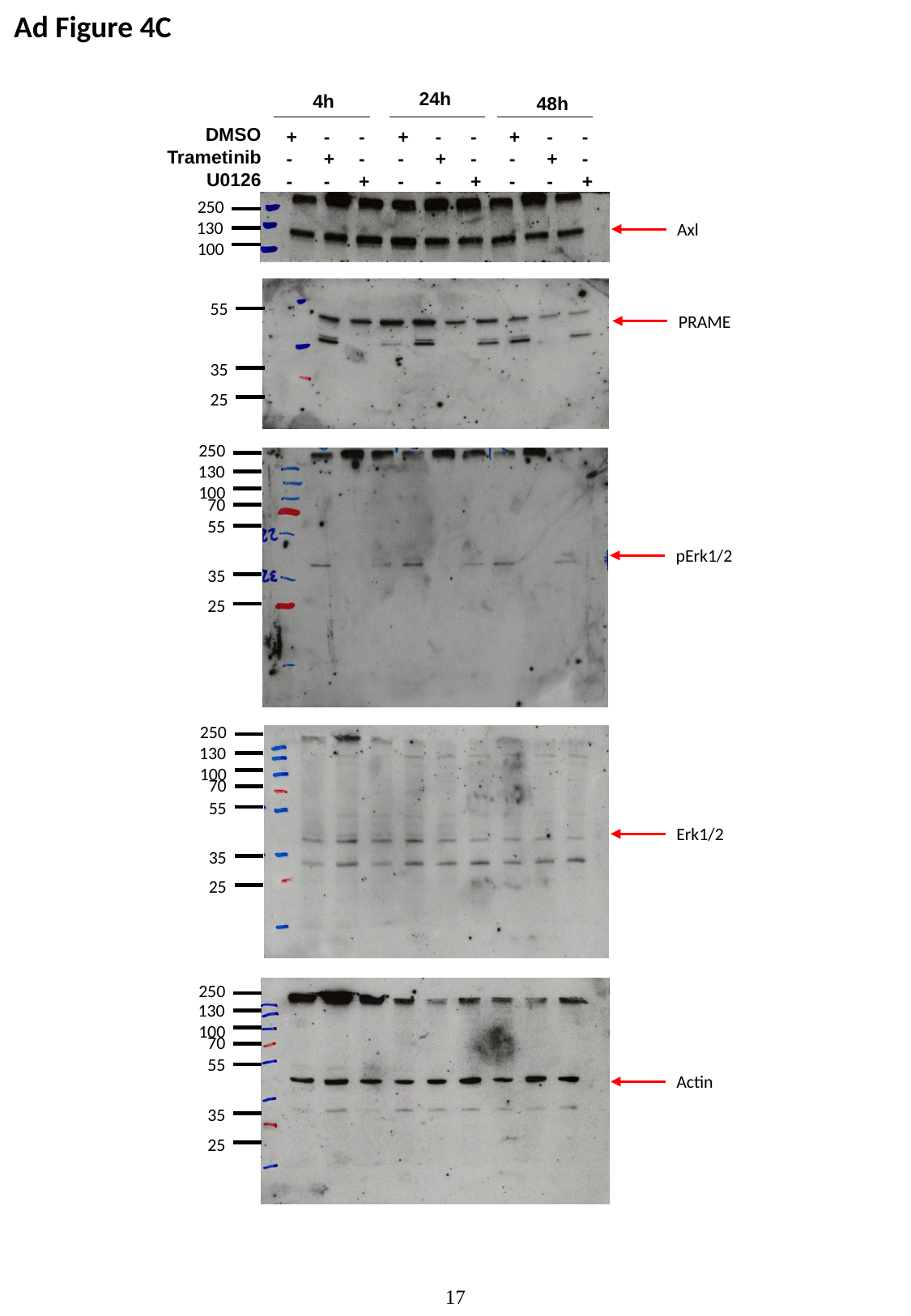

Ad Figure 4C
24h
4h
48h
DMSO
Trametinib
U0126
+
-
-
-
+
-
-
-
+
+
-
-
-
+
-
-
-
+
+
-
-
-
+
-
-
-
+
250
130
100
Axl
55
35
25
PRAME
250
130
100
70
55
35
25
pErk1/2
250
130
100
70
55
35
25
Erk1/2
250
130
100
70
55
35
25
Actin
17

## Slide 18
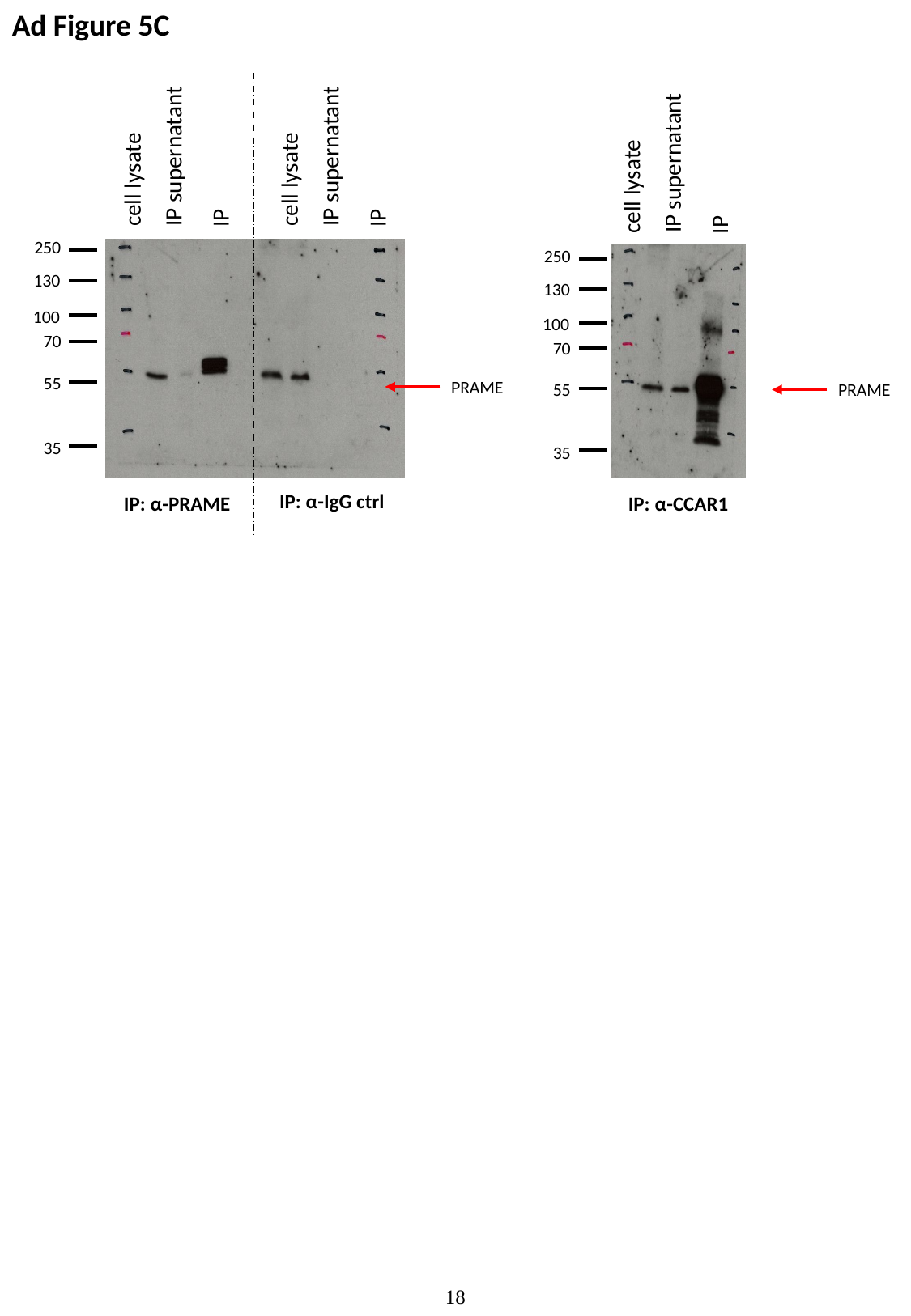

Ad Figure 5C
IP supernatant
IP supernatant
cell lysate
cell lysate
IP
IP
250
130
100
70
55
35
PRAME
IP: α-IgG ctrl
IP: α-PRAME
IP supernatant
cell lysate
IP
250
130
100
70
55
35
PRAME
IP: α-CCAR1
18

## Slide 19
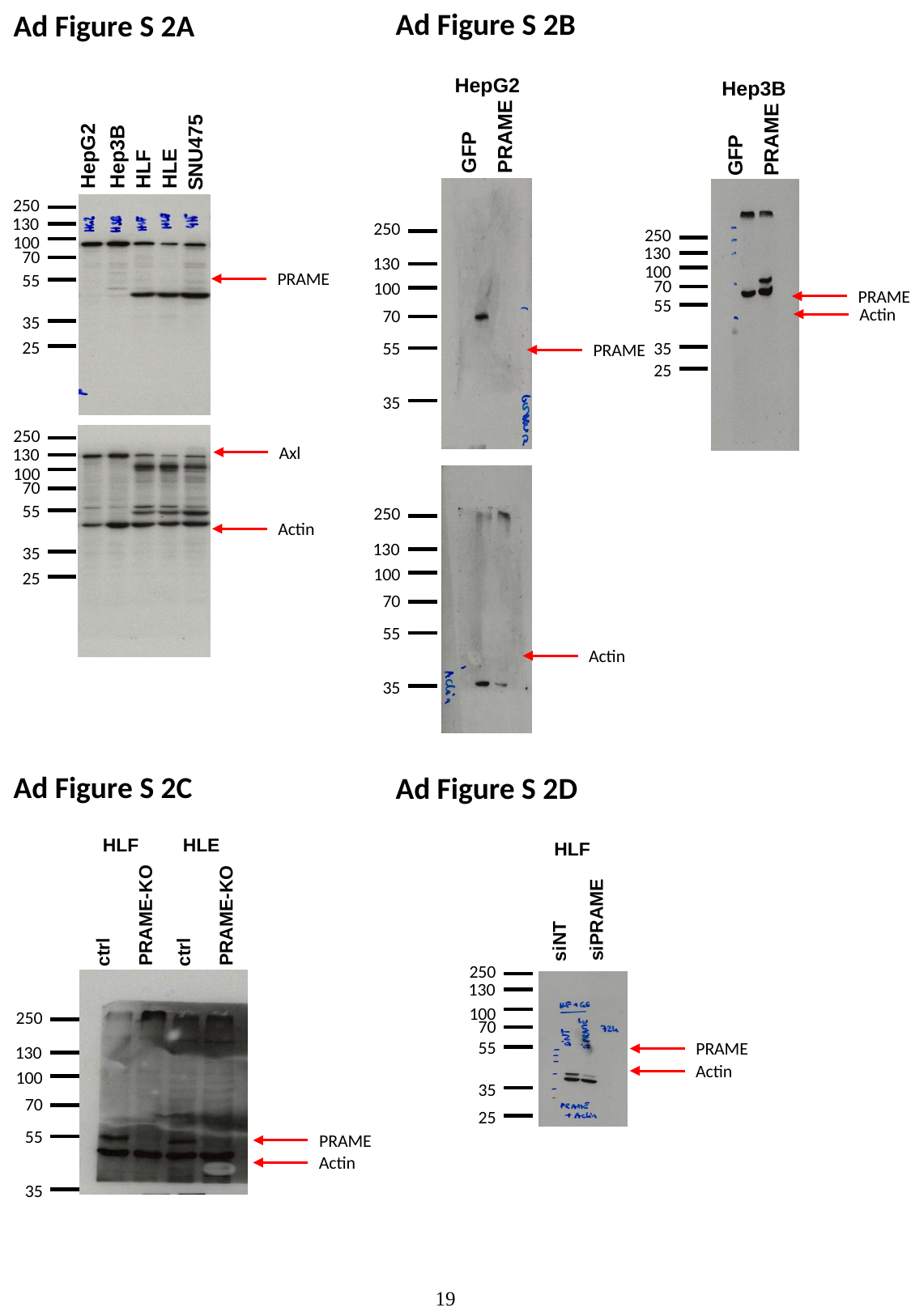

Ad Figure S 2B
Ad Figure S 2A
HepG2
PRAME
GFP
250
130
100
70
55
35
PRAME
Hep3B
PRAME
GFP
250
130
100
70
PRAME
55
Actin
35
25
SNU475
HepG2
Hep3B
HLE
HLF
250
130
100
70
PRAME
55
35
25
250
Axl
130
100
70
55
Actin
35
25
250
130
100
70
55
35
Actin
Ad Figure S 2C
Ad Figure S 2D
HLE
HLF
PRAME-KO
PRAME-KO
ctrl
ctrl
250
130
100
70
55
PRAME
Actin
35
HLF
siPRAME
siNT
250
130
100
70
55
PRAME
Actin
35
25
19

## Slide 20
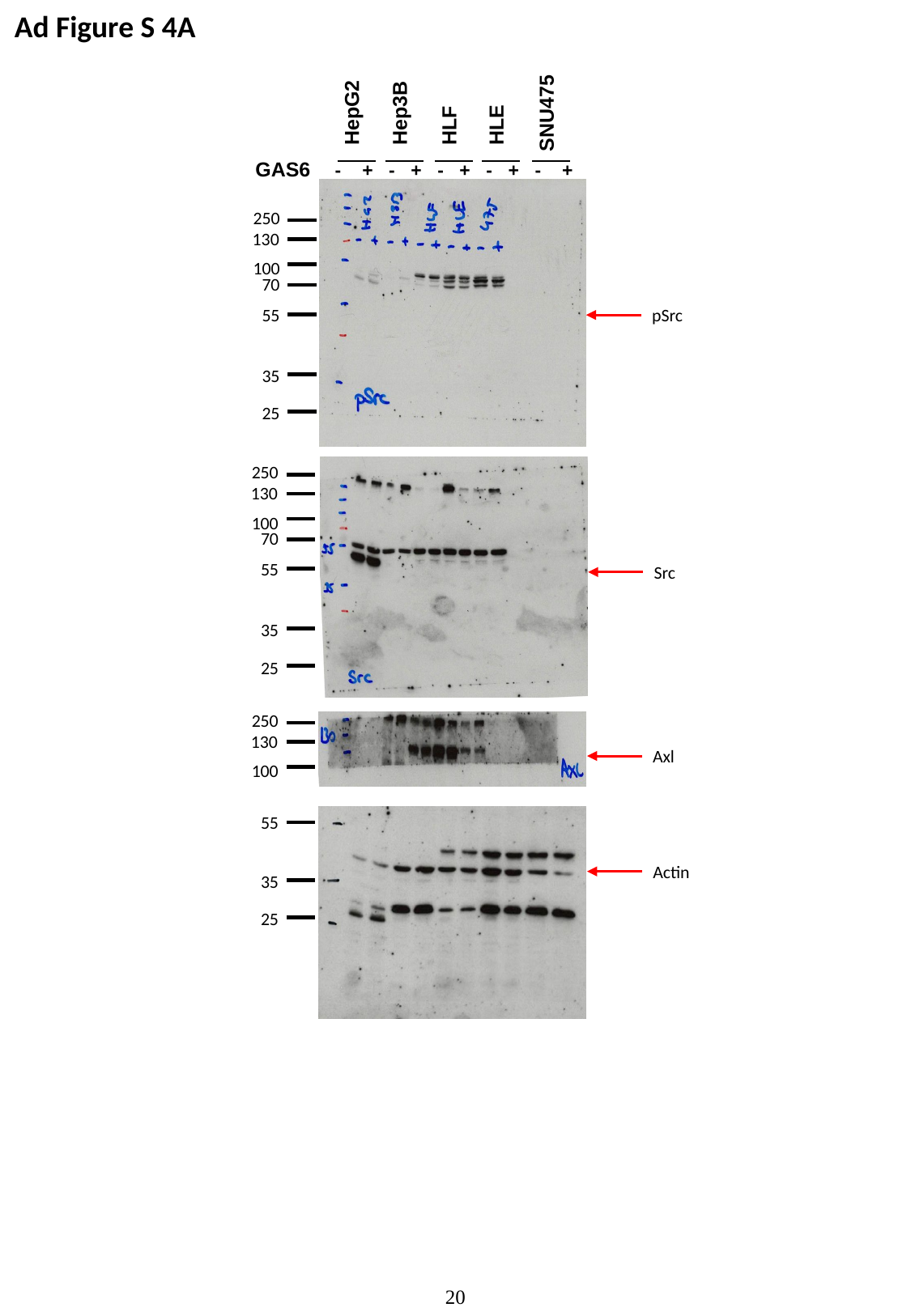

Ad Figure S 4A
SNU475
HepG2
Hep3B
HLF
HLE
GAS6
- + - + - + - + - +
250
130
100
70
55
pSrc
35
25
250
130
100
70
55
Src
35
25
250
130
Axl
100
55
Actin
35
25
20

## Slide 21
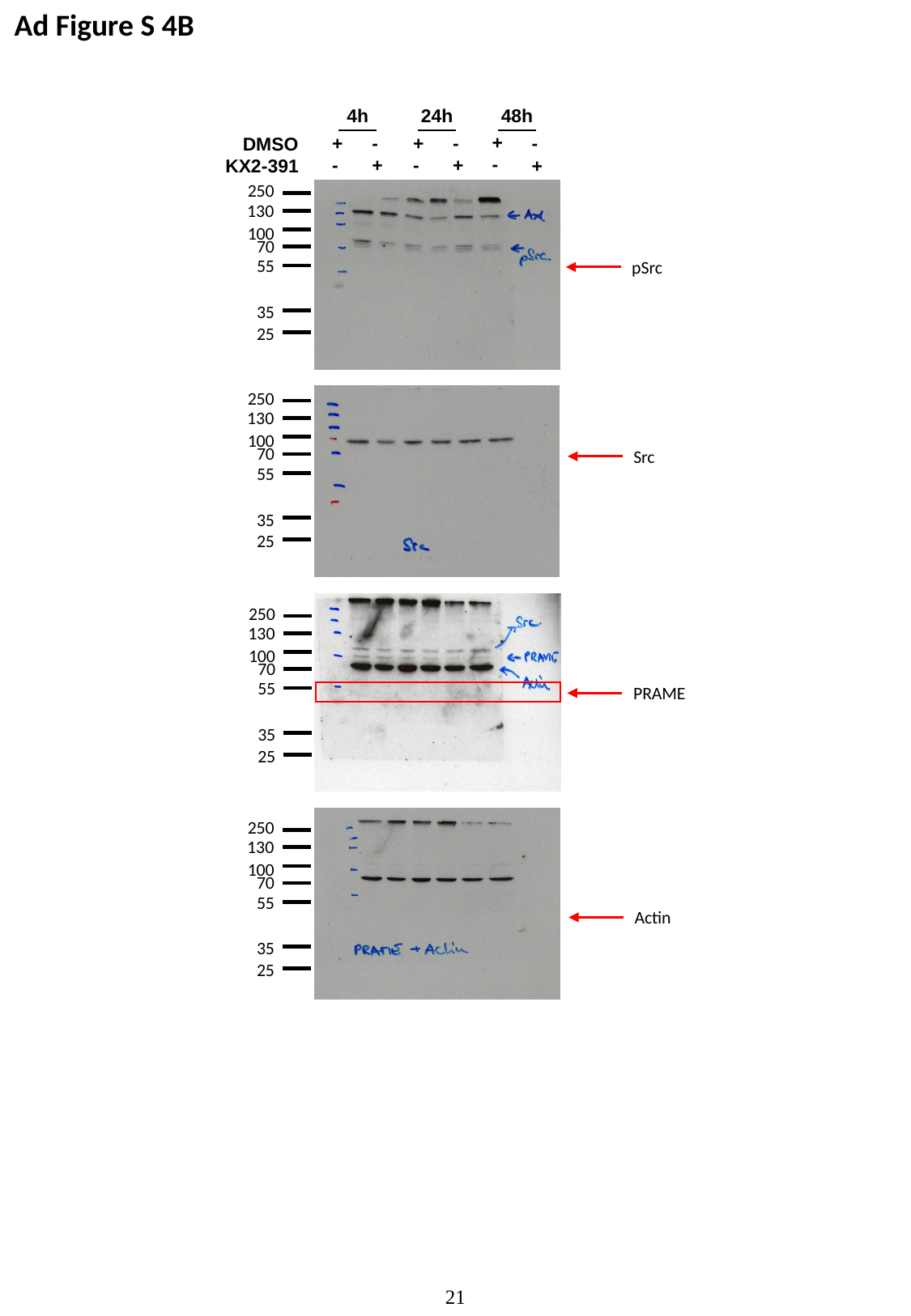

Ad Figure S 4B
4h
24h
48h
+
-
+
-
+
-
-
+
-
+
-
+
DMSO
KX2-391
250
130
100
70
55
pSrc
35
25
250
130
100
70
Src
55
35
25
250
130
100
70
55
PRAME
35
25
250
130
100
70
55
Actin
35
25
21
